# Job satisfaction of nurses with master of nursing degrees in Poland: quantitative and qualitative analysis

**DOI:** 10.1186/s12913-018-3053-6

**Published:** 2018-04-03

**Authors:** Aneta Brayer, Ludmila Marcinowicz

**Affiliations:** 1 Department of Pediatrics, Independent Public Children’s Hospital in Warsaw, Zwirki i Wigury 63A, 02 091 Warszawa, Poland; 2 Department of Primary Health Care, Medical University of Bialystok, Mieszka I 4 B, 15 054 Bialystok, Poland

**Keywords:** Job satisfaction, Nurse, Master degree, Misener nurse practitioner job satisfaction survey

## Abstract

**Background:**

Understanding the issue of job satisfaction of nurses with master of nursing degrees may help develop organisational changes necessary for better functioning of health care institutions. This study aimed to evaluate the level of job satisfaction among holders of Masters of Nursing degrees employed at health care institutions and to ascertain its determinants.

**Methods:**

The cross-sectional study was carried out in randomly selected health care institutions in Poland using the Misener Nurse Practitioner Job Satisfaction Survey and an original survey questionnaire with two open-ended questions. Quantitative data were analysed using descriptive and summary statistics.

**Results:**

The participants gave highest satisfaction ratings to their relationships with direct superiors and other nurses, as well as their social contacts at work. The lowest ratings were given to the pension scheme and factors connected with remuneration. A highly statistically significant relationship was found between the job classification and the level of professional satisfaction (*p <* 0.001). Qualitative analysis of responses to the two open-ended questions supported Herzberg’s Two-Factor theory: internal factors promoted satisfaction, whilst external ones caused dissatisfaction.

**Conclusions:**

Managers of nurses should strengthen the areas that contribute to higher employee satisfaction, particularly interpersonal relationships, by commendation and recognition of work effects.

## Background

Due to variability amongst educational standards and the organisational characteristics of health care systems in different countries, professional nurses are diversified in terms of their education levels. In Poland, as in many other European countries, the nursing education system consists of two stages: a 3-year bachelor’s level course (1st cycle) and a 2-year master’s level course (2nd cycle). The duration of master’s nursing studies is at least 4 semesters; practical classes and traineeships must include at least 1300 h, and the number of European Credit Transfer and Accumulation System (ECTS) points is at least 120 [1]. Advancement to master’s level studies is available for those who have graduated from a 1st cycle nursing course.

Graduates of master’s programmes have specialist knowledge in nursing and other medical sciences. They can solve professional problems (especially those that involve making decisions in difficult situations), establish the standards of professional care and implement them in professional practise, monitor the quality of care, and conduct research. Additionally, they are prepared to organise and supervise nursing care, apply legal regulations in management, determine the assumptions of human resources policy, and plan employment at the medical facility. A master’s-prepared nurse is also qualified to develop and implement health education programmes and select optimum teaching and learning methods [1]. Moreover, a nurse with the Master’s degree may engage in doctoral (3rd cycle) studies.

Medical professionals address problems of quality-management in health care; they influence the hospital’s organisational culture and their colleagues’ behaviours; and they participate in building a positive image of the medical institution, through contacts with patients and their families [2]. Master’s studies help nurses develop the habit of continuous learning, and hence ensure continuous professional development. This is especially important in the nursing profession, with its central focus on caring for people. Nurses with a Master’s degree (hereafter referred to as ‘masters of nursing’) can work at health care institutions, in state or local administration, as teachers of the profession, or as researchers.

The current system of nursing education in Poland has definitely contributed to the increasing number of masters of nursing, and thus to the development of Polish nursing itself, through research into the practise of nursing, comparative studies of nursing experiences in Poland versus other countries, obtaining of higher (e.g. PhD) degrees, and organising of conferences and symposia. Data of the Central Statistical Office [3] show that in 2013, amongst medical professionals working directly with patients in health care centres in Poland, there were 200,587 nurses, of whom 19,920 (9.9%) had Master of Nursing degrees.

Although work satisfaction amongst nurses has been studied by many scholars [4–10], there are still only a few studies concerning the cohort of nurses with Master’s degrees [11, 12]. There is some evidence, however, for positive gains for nurses who undertake postgraduate nursing studies at the master’s level, related to professional and personal qualities which may provide direct benefit to patients [11].

To maximise the knowledge and skills of nursing professionals, it is worth identifying the areas of dissatisfaction of nurses who have Master of Nursing degrees and the factors that contribute to satisfaction with the work they do. Learning about and understanding the issues related to professional satisfaction of this cohort may help develop organisational changes necessary for improved functioning of health care institutions.

The objectives of this work were to evaluate the level of professional satisfaction of nurses with Master of Nursing degrees employed at health care institutions and to ascertain its determinants.

The specific aims were:

1. to determine the general level of professional satisfaction of nurses who have Master of Nursing degrees;
2. to determine the hierarchy of the factors with the greatest and the least influence on professional satisfaction;
3. to determine the relationship between the level of professional satisfaction and the person’s position;
4. to identify the factors of professional satisfaction and dissatisfaction based on the responses to open-ended questions.

## Methods

This cross-sectional study was carried out in randomly selected health care institutions in Poland between October 2013 and March 2014. Participation in the research was anonymous and voluntary. The following inclusion criteria were adopted: having the degree of Master of Nursing (or equivalent designation), currently employed as a nurse, and expressing consent to participate in the study. The questionnaires were distributed amongst all available masters of nursing employed in each hospital during the study (a total of 1073 participants). Six hundred eighty-six correctly and fully completed questionnaires were analysed, a response rate of 64%. The Misener Nurse Practitioner Job Satisfaction Survey (MNPJSS) and an original survey questionnaire were used to collect the data.

The consent of the Bioethics Committee of the Medical University of Bialystok was obtained for the study (Resolution no. R-I-002/310/2013).

### Misener nurse practitioner job satisfaction survey

The MNPJSS was developed in the USA [13] and includes 44 statements referring to different aspects of work. Responses are given in a 6-point Likert scale (1 – the lowest level of satisfaction, 6 – the highest level of satisfaction). Adaptation of the MNPJSS instrument to the Polish setting began by obtaining consent to use the MNPJSS in the present study (consent was provided by De Anna Cox, MN, APRN, FNP-BC, College of Nursing, University of South Carolina, Columbia, SC, USA). The original English version of the MNPJSS questionnaire was translated into Polish. Then, after establishing the final version of the translation, a back-translation to English was applied. Any remaining concerns were settled by group discussion amongst 3 masters of nursing. To test the usability of the MISENER questionnaire under Polish conditions, a pilot study was carried out in 2013 concerning the satisfaction and dissatisfaction of nurses with master’s level education employed at medical institutions. The pilot study involved 272 participants with Masters of Nursing degrees employed at 9 hospitals in central and northeast Poland [14, 15].

### Original survey questionnaire

The original survey questionnaire included 14 questions concerning the workplace, position, specialisation, family and financial situation, age, sex, and working experience. There were also two open-ended questions which allowed the respondents to extemporise: (1) What makes the nursing job satisfying for you? (2) What makes the nursing job dissatisfying for you?

### Analysis

Statistical analysis was performed with the use of Statistica v.13.0. The aggregate score was calculated from the responses and represents a synthesis of each respondent’s opinion units, which can range between 44 and 264 points; 264 points would reflect the maximum level of satisfaction, and 44 the maximum dissatisfaction with work. Quantitative data were analysed using descriptive and summary statistics. i.e., arithmetic mean, median, minimum and maximum values, standard deviation, and the 25th and 75th percentiles. One-way analysis of variance was used to evaluate differences between the groups. It was assumed that *p <* 0.05 was statistically significant.

To improve the validity [16] of the results obtained in the Misener scale, we used the respondents’ answers to two open-ended questions from the original survey questionnaire: (1) What makes the nursing job satisfying for you? (2) What makes the nursing job dissatisfying for you?

Reliability coefficients of the scores were estimated using Cronbach’s alpha coefficient, which assesses internal consistency reliability [17]. The Cronbach’s alpha coefficient for the scale was 0.96.

### Analysis of the answers to the two open-ended questions

All written responses to the two open-ended questions were fully entered into a computer database, but separated based on whether the respondents were satisfied or dissatisfied. Comments to the open questions were studied by content analysis [18]. Because the respondents referred to many aspects at the same time, their responses were divided into components (units). Then, using the technique of content analysis and preserving the division into satisfaction (internal) factors and dissatisfaction (external) factors, they were attributed to factor groups identified and named in accordance with Herzberg’s theory [19].

## Results

### Characteristics of the respondents

The vast majority of the 686 respondents were women (97.5% vs. 2.5% men). The study sample was diverse in terms of age. The largest proportion of respondents ranged in age from 41 to 50 years (41.1%). More than half of the respondents had nursing experience of 21–30 years (35.6%) or 11–20 years (23.6%). Nearly three-fourths (71%) did not have any specialisation. The respondents were employed at different types of hospitals. The highest number worked at clinical hospitals (36%). Two-thirds worked as divisional nurses (66.6%). One quarter was a senior charge nurse or a coordinating nurse (25.7%). Relatively few respondents held higher managerial positions (4.7%). A minority (3.1%) of the participants had other positions, such as epidemiology nurse or endoscopy nurse (Table 1).

**Table 1:** Characteristics of the respondents

Characteristic	*n* = 686	Percent
Gender		
Women	669	97.5
Men	17	2.5
Age (years)		
< 31	150	21.9
31–40	183	26.7
41–50	282	41.1
51–60	70	10.2
61–65	1	0.1
Years of experience in nursing		
< 5	112	16.3
5–10	108	15.8
11–20	162	23.6
21–30	244	35.6
> 30	60	8.7
Specialization		
Yes	199	29.0
No	487	71.0
Type of hospital		
University Clinical Hospital	247	36.0
Provincial	236	34.4
Poviat	203	29.6
Position		
Managerial	32	4.7
Senior charge / Coordinating	176	25.6
Divisional	457	66.6
Others	21	3.1

### Mean rating of each aspect of work

**Fig. 1:**
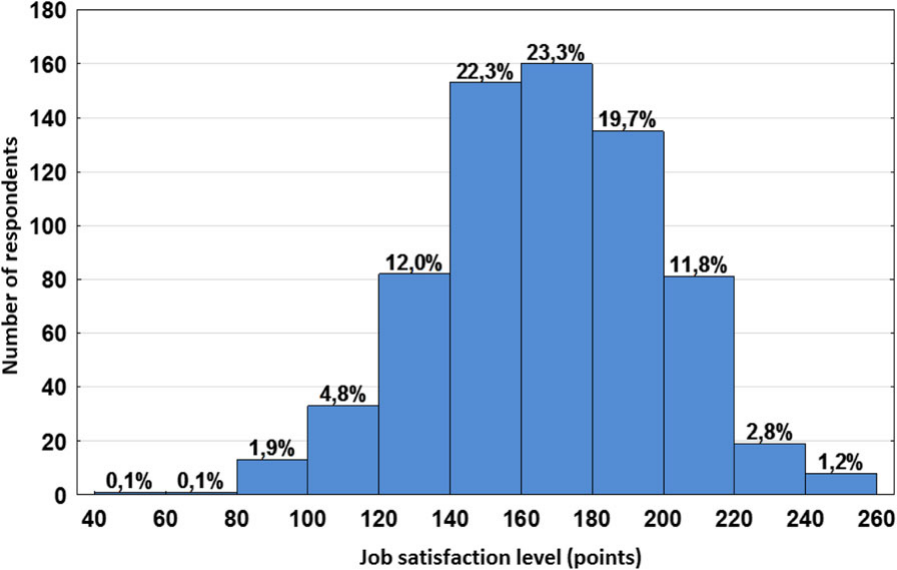
Distribution of aggregate scale of professional satisfaction in the whole study sample

The distribution of the aggregate scale of professional satisfaction in the whole study sample is presented in Fig. 1. The mean value is approximately 168 points, the lowest, 58, and the highest, 260.

Table 2 shows the mean ratings by study participants of all aspects of work, from best to worst. The participants gave the highest ratings to their relationships with direct superiors and other nurses, as well as their social contacts at work. They were also satisfied with the vacation entitlement, the complexity of health problems encountered at work, and the resulting challenges which gave them a sense of professional fulfilment. The lowest ratings were given to the pension scheme and factors connected with remuneration (including cash bonuses, compensation for extra services, and the distribution of awards). Dissatisfaction also resulted from scientific development issues (including the level of engagement in research and insufficient time or financial support for continuing education, time off to serve on professional committees), as well as the status of the nursing profession in the community (i.e. perceived as undervalued).

**Table 2:** Mean rating of each aspect of work

Items	Mean	SD
Immediate supervisor	5.07	0.93
Interaction with other NPs including faculty	4.83	0.84
Social contact at work	4.53	1.04
Vacation/Leave policy	4.50	1.09
Patient mix	4.41	0.97
Challenge in work	4.41	1.02
Sense of value for what you do	4.40	1.09
Recognition of your work from peers	4.38	0.95
Sense of accomplishment	4.29	1.13
Social contact with your colleagues after work	4.27	1.18
Quality of assistive personnel	4.23	1.11
Expanding skill level/procedures within your scope of practice	4.23	1.09
Recognition for your work from superiors	4.08	1.21
Ability to deliver quality care	4.06	1.16
Acceptance and attitudes of physcians outside of your practice	4.05	1.20
Patient scheduling policies and practices	4.03	1.07
Freedom to question decisions and practices	4.03	1.20
Professional interaction with other disciplines	4.02	1.07
Level of autonomy	3.98	1.13
Process used in conflict resolution	3.93	1.12
Respect of your opinion	3.92	1.14
Evaluation proces and policy	3.90	1.14
Time allotted for review of lab and other test results	3.89	1.16
Opportunity to develop and implement ideas	3.88	1.17
Consideration given to your opinion and suggestions for change in the work setting or office practice	3.86	1.18
Amount of consideration given to your personal needs	3.85	1.16
Opportunities to expand your scope of practice and time to seek advanced education	3.77	1.21
Amount of administrative support	3.75	1.25
Opportunity for professional growth	3.73	1.34
Input into organizational policy	3.72	1.17
Opportunity to expand your scope of practice	3.66	1.26
Time allotted for answering messages	3.59	1.33
Benefit package	3.56	1.32
Time allocation for seeing patients	3.52	1.21
Percentage of time spent in direct patient care	3.50	1.25
Flexibility in practice protocols	3.48	1.26
Time off to serve on professional committees	3.28	1.29
Status in the community	3.15	1.33
Support for continuing education (time and money)	3.13	1.42
Amount of involvement in research	3.08	1.34
Reward distribution	2.97	1.46
Opportunity to receive compensation for services performer outside of your normal duties	2.42	1.42
Monetary bonuses that are available in addition to your salary	2.39	1.42
Retirement plan	2.22	1.28

### Professional satisfaction level and the position

The factor that significantly affected the level of professional satisfaction was the position held at work. Participants with managerial positions rated their work satisfaction 10 points higher than senior charge nurses, and senior charge nurses reported having an almost 10-point higher work satisfaction rating compared to divisional nurses. A highly statistically significant relationship was found between the position and the level of professional satisfaction (*p <* 0.001) (Table 3).

**Table 3:** Professional satisfaction level depending on the position

Position	Professional satisfaction level (points)					
	*n* = 686	$\overline{x}$	Me	s	Min	Max
Managerial	32	182.8	180.0	30.8	124	252
Senior charge/coordinating	176	173.3	176.0	30.4	86	259
Divisional	457	164.1	164.0	31.9	58	259
Other	21	182.1	187.0	36.4	124	260
*p*	0.0000***					

***A highly statistically significant

The responses to the open-ended questions were divided into factors based on Herzberg’s theory. Among the 686 respondents, 484 (70.6%) answered the question concerning professional satisfaction, and 421 (61.4%) answered the question concerning the reasons for lack of satisfaction with the job. The respondents’ answers referred to many aspects of work. Therefore, the responses were divided into 1732 units, including 864 factors causing satisfaction (49.9%) and 868 factors causing dissatisfaction (50.1%).

The following categories were identified as external factors: remuneration, working conditions, interpersonal and social relationships, company policy, and professional status. In this group of factors, most answers referred to dissatisfaction (40.7%), for example:

“Very low pay! Especially in comparison with the responsibility connected with the nurse's job”;

“Little respect from patients, their families, and the medical circle Although we gain new skills and improve our qualifications, we cannot use them in our work”.

Internal factors were categorised as follows: accomplishments, development and promotion, the content of work, responsibilities, and recognition. In this group, in contrast to the preceding one, most answers referred to satisfaction (43.3%) (Table 4), for example:

“I find fulfillment in this profession and I'm happy to be able to participate in the process of fighting for the primary human values: life and health”;

“I'm happy when I see a child getting better”;

“I work in a team where the atmosphere is really good”.

**Table 4:** Division of responses to the open-ended question by factors based on Herzberg’s theory

Factors based on Herzberg’s theory		Satisfaction		No satisfaction		Total	
		*N*	%	*N*	%	*N*	%
External hygiene factors	1. Remuneration	9	0.52%	304	17.55%	313	18.07%
	2. Working conditions	4	0.23%	24	1.39%	28	1.62%
	3. Relationships and the social contacts	74	4.27%	47	2.71%	121	6.99%
	4. Company policies	14	0.81%	228	13.16%	242	13.97%
	5. Professional status	6	0.35%	102	5.89%	108	6.24%
Totality of external factors		107	6.18%	705	40.70%	812	46.88%
Internal motivational factors	6. Achievements	114	6.58%	4	0.23%	118	6.81%
	7. Professional growth and promotion	88	5.08%	27	1.56%	115	6.64%
	8. Content of the work	429	24.77%	21	1.21%	450	25.98%
	9. Responsibility	50	2.89%	21	1.21%	71	4.10%
	10. Recognition	69	3.98%	80	4.62%	149	8.60%
Totality of internal factors		750	43.30%	153	8.83%	903	52.14%
Others		7	0.40%	10	0.58%	17	0.98%
Total		864	49.88%	868	50.12%	1732	100.00%

## Discussion

The findings of this original study show that nurses with Master of Nursing degrees are most satisfied with their relationships with direct superiors and other nurses, and with social contacts at work. Other authors have reported similar findings concerning nurses’ professional satisfaction [4, 5, 20]. This concordance may be explained by the fact that both in our analysis and in the other studies the vast majority of the respondents were women. As Lipińska-Grobelny [21] observed when analysing associations between gender and compatibility with the profession, for women, interpersonal relationships were the main area of satisfaction with work.

Our research results also show that masters of nursing who held managerial or autonomous positions (e.g. epidemiology, surgical or endoscopy nurse) are the most satisfied with their work. It has been reported in the nursing literature that a higher level of independence at work may lead to higher satisfaction when carrying out tasks [22]. This is also supported by a Slovenian study, which showed that nurses in leadership positions were more satisfied with their work in comparison to other nurses [23]. Given that, in our study, most of the respondents were female, our findings are consistent with those of Zalewska [24], who observed that for women, job autonomy is conducive to job satisfaction.

The present study involved qualitative analysis of responses to open-ended questions with respect to Herzberg’s motivational theory [19, 25]. According to Herzberg, job satisfaction is affected by two independent categories of factors, i.e. external hygiene factors, and internal motivational ones. The first group of factors refers to the working environment and is relatively independent of the person who does the job (pay, company policy and administration, interpersonal relationships with superiors and colleagues, working conditions, the status of the job). External factors are the main reason for dissatisfaction with work. Internal factors, in turn, refer to personal experiences of the employee connected with the work performed, such as, for example, recognition of professional achievements, development and promotion opportunities, elements demonstrating the intrinsic value of the work [19, 25].

More respondents (70.6%) answered the first open-ended question, which concerned the sources of satisfaction, than the second question, which concerned the reasons for dissatisfaction (61.4%). However, thorough analysis of the respondents’ statements showed that the number of response units indicating dissatisfaction (50.1%) was similar to that for satisfaction (49.9%). In our study, the factors that lead to professional satisfaction correspond to the internal factors identified by Herzberg [25]. Specifically, the content of work was mentioned by one-fourth of the participants (24.77%) as a factor that promoted satisfaction. The source of dissatisfaction reflected in the responses to the open-ended questions was the level of remuneration for nurses’ work (17.55%) (see Table 4).

Content analysis of the responses to the open-ended questions provided more insight into the opinions of masters of nursing concerning professional satisfaction. Examples of this may be the ample punctuation marks used by the respondents (mostly exclamation marks) and comments emphasising the importance of the answers and the problems described. The value of open-ended questions in surveys is also confirmed by other studies [26].

The questionnaire that asks both closed questions and open-ended questions is an example of how quantitative and qualitative data are combined [27]. Employing a primarily quantitatively driven approach in our study and adding a qualitative component to supplement the quantitative survey allows the exploration of deeper or fuller answers to the research questions in order to extract policy relevant results.

## Conclusions

The greatest source of satisfaction for masters of nursing is their relationships with direct superiors and other nurses, and the social contacts at work. The greatest source of dissatisfaction is financial factors such as remuneration, the distribution of financial rewards and benefits, and the pension system. Qualitative analysis of responses to open-ended questions confirmed the assumptions of Herzberg’s theory. Internal factors (e.g. the content of the work) promoted satisfaction, whilst external ones (e.g. remuneration) caused dissatisfaction.

### Implications for nursing management

The results of our study may indicate an important direction of activity for managers, who should be interested in keeping professional medical personnel – masters of nursing – in Poland. The increasing lack of interest in the nursing profession as a career choice, resulting from, amongst other concerns, low remuneration, is a major issue in many countries [28]. Research carried out in 10 European countries shows that about 9% of nurses (from 5% to 17% in different countries) are thinking about leaving the job [29]. Our study results suggest that managers should strengthen those areas that contribute to employees’ higher satisfaction, particularly interpersonal relationships, by commendation and recognition of work effects.

## Abbreviation

MNPJSS: The misener nurse practitioner job satisfaction survey
